# Sensory Neuropeptides and Endogenous Opioids Expression in Human Dental Pulp with Asymptomatic Inflammation: *In Vivo* Study

**DOI:** 10.1155/2015/879126

**Published:** 2015-10-11

**Authors:** Daniel Chavarria-Bolaños, Hector Flores-Reyes, Nelson Lombana-Sanchez, Bernardino Cerda-Cristerna, Amaury Pozos-Guillen

**Affiliations:** ^1^Faculty of Dentistry, Costa Rica University, 1493-3000 San José, Costa Rica; ^2^Endodontics Postgraduate Program, Faculty of Dentistry, San Luis Potosi University, 78290 San Luis Potosi, SLP, Mexico; ^3^Basic Sciences Laboratory, Faculty of Dentistry, San Luis Potosi University, 78290 San Luis Potosi, SLP, Mexico; ^4^Research and Development, Axopod Consultants for Life, 110921 Bogotá, Colombia

## Abstract

*Purpose*. This study quantified the expression of substance P (SP), calcitonin gene-related peptide (CGRP), *β*-endorphins (*β*-End), and methionine-enkephalin (Met-Enk) in human dental pulp following orthodontic intrusion. *Methods*. Eight patients were selected according to preestablished inclusion criteria. From each patient, two premolars (indicated for extraction due to orthodontic reasons) were randomly assigned to two different groups: the asymptomatic inflammation group (EXPg), which would undergo controlled intrusive force for seven days, and the control group (CTRg), which was used to determine the basal levels of each substance. Once extracted, dental pulp tissue was prepared to determine the expression levels of both neuropeptides and endogenous opioids by radioimmunoassay (RIA). *Results*. All samples from the CTRg exhibited basal levels of both neuropeptides and endogenous opioids. By day seven, all patients were asymptomatic, even when all orthodontic-intrusive devices were still active. In the EXPg, the SP and CGRP exhibited statistically significant different levels. Although none of the endogenous opioids showed statistically significant differences, they all expressed increasing trends in the EXPg. *Conclusions*. SP and CGRP were identified in dental pulp after seven days of controlled orthodontic intrusion movement, even in the absence of pain.

## 1. Introduction

Asymptomatic inflammation (AI) (also known as silent, low-grade, or painless inflammation) is a concept that describes a scenario in which an etiologic agent is present without clinical evidence of harm or pain. It can be found in different types of cancer [1], genital tract diseases [2], cerebral infarction [3], or diabetic conditions [4]. The oral environment is also vulnerable to suffer AI under certain pathologies, including chronic apical periodontitis [5] and periodontal disease [4]. Dental treatments such as deep restorations or orthodontic movements [6] can cause incessant injury to dental tissues that are not identified as noxious. Irreversible pulpitis can also occur asymptomatically [7, 8], which may progress to dental pulp necrosis without treatment [9].

Vascular, neural, cellular, and biochemical changes from inflammation can be present in the absence of pain. Neuropeptides released from primary afferent neurons (PAN) are crucial factors in provoking inflammation of neural origin or “neurogenic inflammation” [10]. Substance P (SP) and calcitonin gene-related peptide (CGRP) are capable of inducing vasodilation, plasma extravasation, immune cell chemotaxis, and pain [11, 12]. Controversy exists when both neuropeptides are expressed asymptomatically. This evidence is particularly low in dental pulp studies.

Pain can be modulated by central and peripheral nervous mechanisms. Peripherally, opioid-containing immune cells (OCIC) play the main role due to *β*-endorphins (*β*-End) and methionine-enkephalin (Met-Enk) release [13]. These substances induce the activation of opioid receptors (OR) located in PAN [14], thus causing electrical changes that modulate pain partially via the inhibition of neuropeptide release [15, 16].

Considering both sides, it is interesting to analyze these two families of peptides within dental pulp, using controlled orthodontic forces that may allow nerve response without causing clinical symptomatology [17]. The aim of this study was to determine the expression levels of SP, CGRP, *β*-End, and Met-Enk in human dental pulp following orthodontic intrusion.

## 2. Material and Methods

### 2.1. Patients

Eight patients of both sexes who were between 12 and 16 years old were selected. Patient recruitment was performed at the Endodontics and Pediatric Dentistry Clinics. Inclusion criteria were as follows: being systemically healthy and indicated for the extraction of the four first premolars for orthodontic reasons; teeth without caries, fractures, or periodontal disease; and teeth with radiographically evident complete radicular formation. Exclusion criteria were as follows: patients receiving recent anti-inflammatory, analgesic, or antibiotic treatment; those who had root resorption (from any cause), permanent or provisional restorations in the first premolars or radicular dilacerations; smokers; pregnant patients; and those who have occlusal disorders.

### 2.2. Study Design

A descriptive comparative pilot study was conducted with the approval of the Institutional Ethics Committee (Approval Code number CEIFE-002-010), according to the Declaration of Helsinki. Written informed consent was explained and obtained from parents/legal guardians of each patient. Intrusive orthodontic appliances were designed using standard 0.018′′ orthodontic brackets and double tubes (Sybron/Ormco, Orange, CA, USA), selected for the first premolars and permanent first molars, respectively. Both brackets were bonded to each tooth using the adhesive system Prime & Bond (3M Unitek, Monrovia, CA, USA) and composite Filtek 350 (3M Unitek), standardizing their positions with an Anderson calibrator (Dentaurum GmbH, Ispringen, Germany). Using number 139 orthodontic pliers (Dentaurum GmbH), a 1 mm loop was formed with a retentive fold on the opposite side of a stainless steel wire (0.018′′  ×  0.025′′). The customized wire was placed in the tube slot and adjusted to allow the loop to be bent until an acute angle was achieved. Using a dynamometer (Corex, Haag-Streit, Kowniz, Switzerland), the angulation was standardized at 40° to allow for a 150–200 g intrusive force.

Two premolars from each patient were randomly selected and assigned to two different groups: the asymptomatic inflammation group referred to as experimental group, which would undergo controlled intrusive force for seven days (EXPg), and the control group (CTRg), used to determine the basal levels of each substance. Each patient was scheduled to obtain the samples from the control tooth before any clinical intervention took place. Each control tooth was anesthetized with 4% prilocaine (Pricanest, Ropsohn Therapeutics, Bogotá, Colombia). From that moment, the entire sampling procedure was performed within a 10-minute period. After extraction, a second assistant created a 1 mm longitudinal groove over the vestibular tooth surface using a high-speed diamond bur with copious irrigation to facilitate the mechanical fracture of the tooth and the intact acquisition of pulpal tissue, which was immediately stored in a tagged cryovial containing 1.8 mL of 4% paraformaldehyde. The cryovial was stored at −70°C. The orthodontic device assigned to the EXPg was then placed. After its activation, each patient received instructions to call if any problem or severe discomfort was experienced and, if necessary, to take 400 mg of ibuprofen as a rescue analgesic.

For the following six days, the patients were contacted by telephone to evaluate their progress along the experimental period. Information on their symptoms, analgesic usage, or orthodontic device displacement was recorded. At day seven, patients were scheduled to obtain the dental pulp sample from EXPg, as described above.

### 2.3. Radioimmunoassay

A radioimmunoassay (RIA) was performed to quantify the amount of each substance obtained from each sample, according to previous investigations reported [18]. SP, CGRP, *β*-End, and Met-Enk release were determined by competition RIA binding assays using a human SP RIA-kit (reference RK-061-05), a human CGRP RIA-kit (reference RK-015-02), a human *β*-End RIA-kit (reference RK-022-14) (Phoenix Peptide Pharmaceuticals, Burlingame, CA, USA), and a human Met-Enk RIA-kit (reference S-2119) (Peninsula Laboratories LLC, Bachem Group, San Carlos, CA, USA). For each kit, 100 *μ*L of antiserum and 100 *μ*L of various neuropeptide/opioid concentrations (1–128 pg *μ*L^−1^) or 100 *μ*L of dental pulp tissue extracts were incubated in polypropylene tubes at room temperature for 20 hours. Then, 100 *μ*L of radioactive 125I tracer was added and left to incubate for another 24 hours. Bound fractions were precipitated by the addition of 100 *μ*L of a secondary antibody (goat anti-rabbit immunoglobulin G serum), 100 *μ*L normal rabbit serum, and 500 *μ*L RIA buffer containing 1% polyethylene glycol 4,000. After 2 hours of incubation at room temperature, the suspensions were spun at 3,500 rpm (4,000 g) for 40 minutes at 4°C to precipitate the bound fractions. The supernatants were carefully aspirated, and pellet radioactivity was read on a gamma counter (Model B5002, Packard Instrument International, Zurich, Switzerland). All samples were assayed in duplicate, and the mean values were calculated. Finally, Scatchard analysis of the binding data was used to assess the amount of neuropeptide/opioid present in each sample.

### 2.4. Statistical Analyses

The results were analyzed by the Mann-Whitney *U* test to compare the differences among groups for continuous variables. A difference was considered to be significant if the probability of its occurring by chance alone was <5% (*P* < 0.05) in a two-tailed test.

## 3. Results

The results are shown in Table 1. In CTRg basal levels of both neuropeptides and endogenous opioids were observed. In the EXPg, SP and CGRP exhibited statistically significant different levels (*P* < 0.05). Although none of the endogenous opioids showed statistically significant differences (*P* > 0.05), they all expressed increasing trends in the EXPg. None of the patients enrolled were eliminated from the study because no one reported severe pain episodes or the need to take the rescue analgesic treatment. All patients reported tolerable discomfort localized in the tooth assigned to EXPg, for the two or three days after orthodontic activation. By day seven, all of the symptoms were absent, and all of the orthodontic devices were still active.

## 4. Discussion

Mechanisms related to neurogenic inflammation and pain modulation in AI are not fully understood. In this study, the expression of two somatosensory neuropeptides and two OPs in dental pulp AI caused by orthodontic intrusion was determined. This clinical model allowed us to obtain a biphasic inflammatory transition, which began with discomfort during the acute phase and finally achieved an asymptomatic state [6]. The intrusion rate of the teeth was not evaluated clinically. Since the intrusion period of the experiment was seven days, it was considered not objective to make a clinical measurement of the distance. In this experiment, intrusion stimulus was only measured in function of time. Although the majority of available evidence is focused on the biochemistry of painful conditions, the study of AI is a promising field that may help explain how important diseases appear after the development of  “anonymous” inflammation. In this study, the presence of an initial inflammatory tenderness was required to assess the beginning of the asymptomatic phase; it was a requirement for this study that all patients report no presence of pain when the samples were obtained.

**Figure 1:**
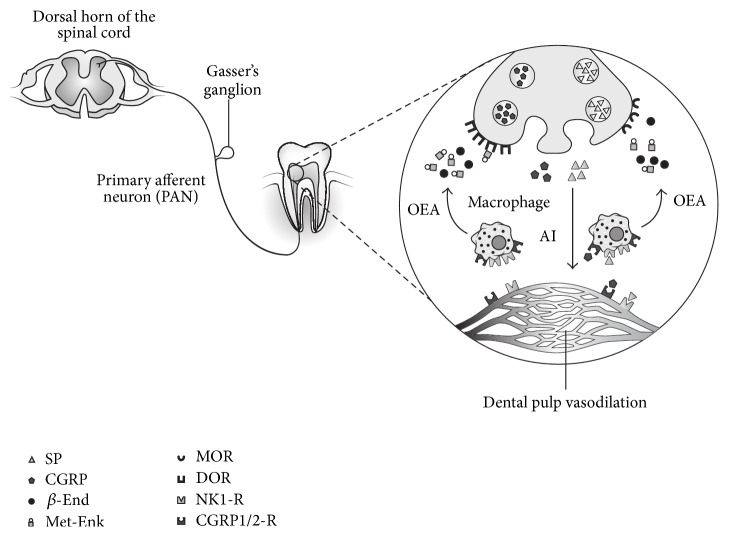
Proposed relationship between neurogenic inflammatory and endogenous opioid analgesic mechanisms. SP: substance P, CGRP: calcitonin gene-related peptide, *β*-End: *β*-endorphin, Met-Enk: methionine-enkephalin, NK1-R: neurokinin 1 receptor, CGRP1/2-R: calcitonin gene-related peptide receptors 1 and 2, MOR: *μ*-opioid receptor, DOR: *δ*-opioid receptor, OEA: opioid endogenous analgesia, AI: asymptomatic inflammation, and PAN: primary afferent neuron.

Orthodontic movements are able to induce the local release of SP and CGRP. Previous studies have demonstrated, within dental pulp, the expression of markers of neurogenic inflammation (substance P and CGRP) using the model of inflammation employed in this study; this controlled orthodontic movement was selected as a well-standardized available model for human clinical trials focused on inflammation [6, 19–21]. Chemotaxis of the macrophage population induced by neuropeptides in late orthodontic inflammation [22] is crucial to establish the neuropeptidergic and OP relationship. Furthermore, nerve fiber sprouting and neurogenic inflammation are common during pulpal insult, including early force application during orthodontic tooth movement [23]. Macrophages express NK1 and CGRP1/2 receptors, which are activated by SP and CGRP, respectively [11], favoring their activation [15, 24]. These cells are the main secretors of OP [25], including *β*-End and Met-Enk [26]. Interestingly, under inflammatory conditions, PAN, the main source of local SP and CGRP release [14], exhibits an upregulation expression of OR [13, 27], which can be targeted by macrophage analgesic products (Figure 1). Controversy surrounds the fact that macrophages are also capable of secreting somatostatin (a substance able to cause analgesia); thus, pain control may not be exclusively caused by OP. However, the local presence of SP selectively inhibits somatostatin release but not *β*-End [24].

These results demonstrate an important increase of SP and CGRP levels in EXPg (*P* < 0.05). The release of sensory neuropeptides occurs almost immediately, in a manner described as an “axonal reflex” [22]. Such a response supports the role of neurogenic inflammation of the dental pulp after low-grade orthodontic continuous movement [20, 22, 28]. Both neuropeptides are frequently associated with pain from pulpal origin [29–31]; however, they have also been identified in asymptomatic irreversible pulpitis [32]. The importance of the role of neuropeptides in inflammation was previously studied by determining their absence, not their presence. Different denervation experiments showed how the deprivation of the sensory nerve supply can attenuate the local inflammatory response [9].

*β*-End and Met-Enk showed an increase for EXPg. Both OPs are postulated to play the leading role in endogenous antinociception [33] due to their action as negative regulators of neurogenic inflammation [16] by the inhibition of SP and CGRP release [14, 15]. However, this study shows that, after orthodontic intrusion, low levels of OP were not enough to attenuate neuropeptide expression.

It is important to consider why late neurogenic inflammation can develop in the absence of pain. Current evidence offers possible explanations. First, dental pulp is able to adjust slowly to low-grade inflammation, assimilating higher levels of proalgesic mediators in the absence of pain [9]. Second, experimental tooth movement is capable of activating central trigeminal pain modulation mechanisms [34], causing a reduction in the local recruitment of OCIC to peripheral injured tissues; thus, the net effect will be the control of pain and lower peripheral opioid peptide concentration [15, 25, 35–37]. Third, new evidence explores the dual effects of neuropeptides in relation to pain experience. SP enhances opioid release, and it is possible that its N-terminal fragment may act as a miu-opioid receptor (MOR) agonist once its expression is upregulated during inflammation. Both observations may explain the lack of effectiveness of NK1 antagonists to modulate pain [38]. For CGRP, the antinociceptive effect is related to the upregulation of MOR when it is applied centrally [38] and peripherally by the local suppression of interleukin-2 production, causing an anti-inflammatory effect [28]. Finally, though both neuropeptides are strong vasodilators, this effect may be masked by low blood flow after orthodontic intrusion, thus avoiding the increase of inner pressure in a low compliance pulpal environment [39–41]. All of these hypotheses must be evaluated in further studies, including more patients in order to confirm these findings, as well as new complementary experiments including pulp tissue immunostaining to evaluate neurons, peptides activity, and immune and inflammatory cells participation. Also, it is necessary to include peptides measurement at different time points in order to elucidate the possible mechanism of asymptomatic inflammation.

## 5. Conclusions

In this preliminary report, SP and CGRP were identified in dental pulp after seven days of controlled orthodontic intrusion movement, even in the absence of pain. At the same time, endogenous opioids exhibited no statistical differences compared with control levels; however, an increase tendency was appreciable for both.

## Figures and Tables

**Table 1:** Expression levels for substance P (SP), calcitonin-gene related peptide (CGRP), *β*-endorphins (*β*-End), and methionine-enkephalin (Met-Enk) in the control group (CTRg) and the asymptomatic inflammation group (EXPg).

Group	Neuropeptides/endogenous opioids			
	SP	CGRP	*β*-End	Met-Enk
CTRg	83.51 ± 11.35	13.73 ± 2.84	12.44 ± 1.74	2.29 ± 0.84
EXPg	145.93 ± 119.26	22.46 ± 3.06	16.51 ± 8.95	10.74 ± 13.78
*P* value	<0.05^*∗*^	<0.05^*∗*^	>0.05	>0.05

Data are presented in picograms of each substance per milligram of dental pulp simple.

^*∗*^Statistically significant difference.
